# Soft-Template-Based Manufacturing of Gold Nanostructures for Energy and Sensing Applications

**DOI:** 10.3390/bios14060289

**Published:** 2024-06-03

**Authors:** Tushar Kanti Maiti, Wanli Liu, Asghar Niyazi, Adam M. Squires, Sujay Chattpoadhyay, Mirella Di Lorenzo

**Affiliations:** 1 Department of Chemical Engineering and Centre for Bioengineering and Biomedical Technologies (CBio), University of Bath, Claverton Down, Bath BA2 7AY, UK; tushar_m@pe.iitr.ac.in (T.K.M.); an993@bath.ac.uk (A.N.); 2 Department of Polymer and Process Engineering, IIT Roorkee, Saharanpur 47001, India; sujay@pe.iitr.ac.in; 3 Department of Chemistry, University of Bath, Claverton Down, Bath BA2 7AY, UK; wl443@bath.ac.uk (W.L.); as3474@bath.ac.uk (A.M.S.)

**Keywords:** nanostructured gold, glucose fuel cell, soft-template metal electrodeposition

## Abstract

Implantable and wearable bioelectronic systems can enable tailored therapies for the effective management of long-term diseases, thus minimising the risk of associated complications. In this context, glucose fuel cells hold great promise as in- or on-body energy harvesters for ultra-low-power bioelectronics and as self-powered glucose sensors. We report here the generation of gold nanostructures through a gold electrodeposition method in a soft template for the abiotic electrocatalysis of glucose in glucose fuel cells. Two different types of soft template were used: a lipid cubic phase-based soft template composed of Phytantriol and Brij^®^-56, and an emulsion-based soft template composed of hexane and sodium dodecyl sulphate (SDS). The resulting gold structures were first characterised by SAXS, SEM and TEM to elucidate their structure, and then their electrocatalytic activity towards glucose was compared in both a three-electrode set-up and in a fuel cell set-up. The Phytantriol/Brij^®^-56 template led to a nanofeather-like Au structure, while the hexane/SDS template led to a nanocoral-like Au structure. These templated electrodes exhibited similar electrochemical active surface areas (0.446 cm^2^ with a roughness factor (RF) of 14.2 for Phytantriol/Brij^®^-56 templated nanostructures and 0.421 cm^2^ with an RF of 13.4 for hexane/SDS templated nanostructures), and a sensitivity towards glucose of over 7 μA mM^−1^ cm^−2^. When tested as the anode of an abiotic glucose fuel cell (in a phosphate-buffered solution with a glucose concentration of 6 mM), a maximum power density of 7 μW cm^−2^ was reached; however the current density in the case of the fuel cell with the Phytantriol/Brij^®^-56 templated anode was approximately two times higher, reaching the value of 70 μA cm^−2^. Overall, this study demonstrates two simple, cost-effective and efficient strategies to manipulate the morphology of gold nanostructures, and thus their catalytic property, paving the way for the successful manufacturing of functional abiotic glucose fuel cells.

## 1. Introduction

The effective management of chronic conditions demands the development of bioelectronics that can assist patients and help towards the delivery of personalised therapies [1]. Glucose fuel cells (GFCs) can play a pivotal role as a miniaturised power source for these bioelectronics and/or as a self-powered monitoring tool for a key metabolite, such as glucose [2,3]. As such, there is great interest in the development of abiotic electrocatalysts for GFCs that can advance the progress of this technology towards practical applications by overcoming key issues, such as electrochemical performance, stability, and, in the case of sensing purposes, sensitivity towards the target analyte [4]. Metal-based nanomaterials have been widely explored as electrocatalysts in GFCs due to their chemical stability, durability, cost-effectiveness, catalytic activity and manufacturing simplicity [5,6]. In particular, Au-based nanomaterials have shown great catalytic activity towards the electrooxidation of glucose in neutral pH conditions, which makes them the ideal anodic catalyst material for implantable and wearable GFCs. The catalytic activity of Au-based materials is significantly influenced by factors such particle size, morphology and architecture, which have an impact on the distribution and nature of their catalytic active sites, ultimately influencing their overall catalytic behaviour [7,8]. Irregular gold nanoparticles (NPs) exhibit superior performance compared to their spherical counterparts, due to a higher surface density of gold oxide on the particles, which serves as a rapid redox mediator, facilitating the oxidation of glucose and enhancing the overall catalytic activity towards glucose [9,10]. An enzyme-free glucose sensor utilizing Au nanoparticles was developed with significantly high surface roughness, which exhibited notable catalytic activity for the oxidation of glucose in a phosphate buffer (PBS) solution with a glucose sensitivity of 1.13 μA mM^−1^ cm^−2^ [11]. Furthermore, porous gold films directly electrodeposited onto gold electrodes have been reported, with a three-dimensional foam-like structure, a diverse range of pore sizes and a surface roughness that is nearly a thousand times greater than polished gold electrodes, leading to high current densities during the oxidation of glucose [12,13]. Du Toit et al. have designed porous Au microelectrodes through electrodeposition using a hydrogen bubble template; the resulting porous Au microelectrodes demonstrated outstanding sensitivity to glucose, equal to 3.1 μA mM^−1^ cm^−2^ [14]. In a recent study, abiotic GFCs on a printed circuit board (PCB) were developed using a gold nanostructured anode [3]. While the hydrogen bubble template electrodeposition method has proven to be very effective in the formation of nanostructured Au electrodes, controlled porosity and morphology are difficult to obtain. A solution to this challenge would be the use of a sacrificial templating method. Sacrificial templating methods can be classified as (i) colloidal templating methods, which use colloidal templates for the production of structuresa porous network; (ii) hard-templating methods, where the structure-directing agent is an inorganic material; (iii) soft-templating methods, where organic molecules are used as the directing agent. The colloidal templating method, for example, has been effectively adopted to produce networks of interconnected platinum (Pt) nanowires, which were achieved by passing reducing reagents through mesoporous silica MCM-48 that enables the reduction of the Pt precursors [15,16]. While effective, this templating method may involve a complex series of multi-steps. Furthermore, irregularly shaped mesoporous metals are frequently obtained due to challenges in controlling the metal deposition reactions within the templates [17,18]. In both the hard and soft-template processes, metal solutions are mixed with the template materials, whereby the metal nanoparticles orient themselves to adopt the same structure as the template. The removal of the hard templates involves the use of caustic reagents, which may leave undesired residue in the final mesoporous network [19,20]. Soft-templating methods typically offer smaller reaction environments for metallization, utilizing the self-assembly of amphiphilic molecules, which allows for more precise control over the structure formation [21,22]. Furthermore, after the metallization process, any remaining soft template material can be easily eliminated with a volatile organic solvent [23,24]. The utilization of soft-templates in combination with electrodeposition allows for the generation of intricate metal nanostructures; the electrodeposition process enables direct metallization at room temperature, providing the flexibility to control the rate of electrodeposition based on the properties of the soft -template, while the desired features of the soft-template are effectively captured [23,25]. Attard et al. [26] reported the creation of two-dimensional hexagonal mesoporous Pt thin films by electroplating metal ions dissolved in lyotropic liquid crystals (LLCs), which served as a viscous soft-template. The resulting films displayed a well-organized arrangement of hexagonal mesopores, each with a diameter of less than 3 nm, and by replacing short surfactants with block copolymers, the pores were enlarged to an approximate diameter of 15 nm [25]. Akbar et al. reported the use of phytantriol lipid cubic phases to make three-dimensional Pt films [27] and the addition of Brij^®^-56 to increase the pore size [28].

Recently, the use of micelle structures has been explored as soft–templates, wherein metal ions form weak bonds with the micelles and are electrochemically deposited, thus leading to highly organized and nanoporous film structures that mirror the size of the micelles [29,30]. This method has shown impressive consistency in producing nanoporous metals, including platinum, gold, palladium, copper, nickel, and gold-nickel alloys, with a controlled pore size within the range of 10 to 60 nm [30,31,32,33]. Nevertheless, despite their promise to achieve uniform nanoporous structures, these methods still need to be further refined for pore sizes larger than 100 nm, which may be crucial to meet target catalytic requirements. 

In this study, we demonstrate the generation of gold nanostructured electrodes via two soft-template approaches: (1) electrodeposition within a cubic phase lyotropic liquid-based structure and (2) electrodeposition within an oil-in-water micelle structure. For the first approach, a cubic phase lyotropic liquid crystal template consisting of an interconnected three-dimensional network of hydrophobic and water phase channels of a lipid (phytantriol) and non-ionic surfactant (Brij^®^-56) was considered. The resulting electrode was characterised by an hyperbranched nanofeather-like configuration. The second approach involved the use of a hexane/SDS micelle solution as the template, which resulted in a nanocoral-like structure. The two templated electrodes were fully characterised and the electrochemical activity towards glucose assessed and compared. The electrodes were also tested as the anode of an abiotic glucose fuel cell and the performance was compared with a control anode obtained by gold eletrodeposition but without a template. Overall, this study demonstrates the effectiveness of these two soft-template strategies for the generation of innovative gold nanostructures for GFC and sensing applications. 

## 2. Materials and Methods

### 2.1. Materials

All chemicals utilized in this study were of analytical grade and used without further purification. Hydrogen tetrachloroaurate (III), glucose, and ethanol were purchased from Fisher. Ammonium chloride, sodium phosphate monobasic (NaH_2_PO_4_), sodium phosphate dibasic (Na_2_HPO_4_), sodium chloride, sulfuric acid, and Brij^®^-56 (C₃₆H₇₄O₁₁) were purchased from Sigma-Aldrich. Phytantriol (C_20_H_42_O_3_), with a purity greater than 98.3%, was generously provided by Adina Cosmetics Ingredients, UK. Deionized and double-distilled water, which underwent further purification via a Milli-Q water purification system (Merck Millipore, Watford, UK), was used to prepare all aqueous solutions. A phosphate buffer (PB) solution (0.1 M, pH 7.4) was prepared from sodium phosphate dibasic and sodium phosphate monobasic in Milli-Q water. Glucose was added to the buffer at the specified concentration.

### 2.2. Generation of the Gold Nanostructured Electrodes

**Figure 1:**
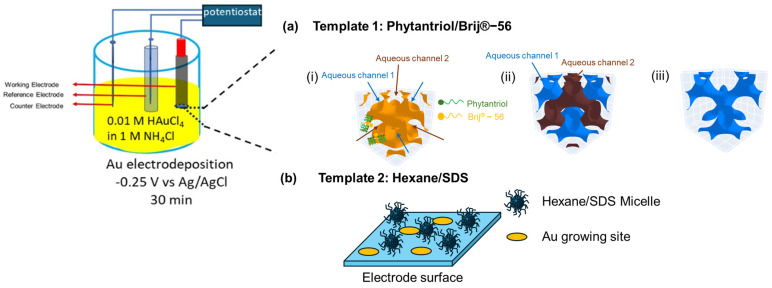
Methodology adopted for the electrodeposition of gold nanostructures with two soft-templates. (**a**) Growing Au nanostructure using the Phytantriol/Brij^®^−56 template: (**i**) a $Q_{II}^{D}$ triply periodic minimal surface formed by lipid membrane of the phytantriol/Brij^®^−56 template (shown as orange) in excess electrolyte solution at the electrode surface; (**ii**) two sets of 4−way non-intersecting aqueous channels separated by the lipid membrane are formed (in blue and brown), with the aqueous electrolyte confined within these channels; (**iii**) the electrodeposition process only takes place through one of the channels that is closer to the bulk (in blue). (**b**) Growing Au nanostructure using the hexane/SDS microemulsion template: randomly dispersed inverse micelles in the electrolyte regulate the diffusion routes of Au ions to reach the electrode surface during electrodeposition processes, leading to specific Au growing sites at the electrode surface.

Prior to use, Au disk electrodes (∅ = 2 mm) were polished with two grades of Al_2_O_3_ powder (0.3 µm and 0.1 µm) on wet polishing cloths, followed by sonication in water for 5 min, and subsequently in EtOH for another 5 min. Two soft-templates were used for gold electrodeposition onto the Au disk electrode, as shown in Figure 1: a liquid crystal-based template and a micelle-based template. For the lipid cubic phase template, individual solutions of Phytantriol and Brij^®^-56 were prepared in ethanol at a weight ratio of 1:1 each. Subsequently, the Phytantriol solution and the Brij^®^-56 solution were mixed in a glass vial with a weight ratio of Phytantriol to Brij^®^-56 to ethanol of 40:10:50. The Au disk electrodes were immersed in this solution for a duration of 10 to 12 min. Following immersion, the Au disk electrodes were left to air-dry at room temperature for 45 min, which led to the concurrent formation of a thin coating comprising of Phytantriol and Brij^®^-56 (Phytantriol to Brij^®^-56 weight ratio of 80:20 in the soft-template coating). Once dried, the electrode was immersed in an electrochemical cell containing 0.01 M HAuCl_4_ in 1 M KCl and operated as the working electrode in a three-electrode set-up, where platinum flag was used as the counter electrode and a Ag/AgCl electrode was used as the reference electrode. The electrodes were connected to a potentiostat (EmStat3+ PalmSens, Houten, The Netherlands). Gold electrodeposition was performed at room temperature at a potential of −0.25 V versus Ag/AgCl for 30 min. Afterwards, the electrodes were washed by immersing in ethanol for 15 min, air dried, and stored at room temperature until used.

For the preparation of the micelle-based template, 3 mL hexane, as the hydrophobic phase, and 0.18 g of sodium dodecyl sulphate (SDS), as the emulsifying agent, were added to 20 mL of a 1 M KCl solution. Then, 0.102 g of HAuCl_4_ was added to the solution. Subsequently, the solution underwent ultrasonication for a duration of 20 min and was then stored for at least 60 min at ambient temperature prior to use. Then, the polished Au disk electrode (geometric surface area = 0.0314 cm^2^) was immersed in the resulting emulsion for 45 min. Gold electrodeposition was carried out using a multi-step amperometry technique with an applied potential of −0.25 V versus Ag/AgCl for 30 min.

A control electrode was prepared in which no template was used for the deposition of gold; in this case, after polishing, the Au disk electrode was immersed in the electrochemical cell containing the aqueous solution of 0.01 M HAuCl_4_ in 1 M KCl, and gold electrodeposition was performed as described above.

The two templated electrodes were analysed using various techniques, including scanning electron microscopy (SEM; Hitachi SU3900, Tokyo, Japan), small-angle X-ray scattering (SAXS; Anton Paar SAXS Point 2.0, Ashland, VA, USA), and transmission electron microscopy (TEM; Jeol 2100Plus, Tokyo, Japan). 

Liquid Crystal Templates composed of Phytantriol and Brij^®^-56 were prepared for SAXS analysis, following the procedure outlined by Akbar et al. [34,35]. Three samples were prepared in glass vials, which consisted of phytantriol and ethanol at a weight ratio of 50:50 (Vial-I); Brij^®^-56 and ethanol at a weight ratio of 50:50 (Vial-II); and the solutions of Vial-I and Vial-II at a weight ratio of 80:20 (Vial-III). The solution from Vial-III was first introduced into Kapton capillary tubes (outer diameter of 1.9 mm), and quickly ejected to leave layers of lipidic coating inside. The coated capillaries were dried at room temperature for 2 h for ethanol evaporation prior to being filled with a solution containing 0.01 M HAuCl_4_ (Au(III)) in 3 M KCl, leading to a set of experiments referred to as Phytantriol/Brij^®^-56/Au(III), or being filled with water, leading to Phytantriol/Brij^®^-56/water. As a control, the solution from Vial-I was also introduced into polyamide capillary tubes filled with water, and referred to as Phytantriol/water. Once filled, the capillary tubes were sealed and the solution was stored for a minimum of 60 min before SAXS measurements. The 2D SAXS patterns of the samples were collected on a Dectris Eiger detector from an Anton Paar SAXS Point 2.0, using Cu source K_α_ radiation (λ = 1.54 Å), with a beam diameter approximately 1 mm, a sample-detector distance of 576 mm, and an acquisition time of 10 min for each sample. The data reduction of 2D to 1D radial profiles was performed using Anton Paar SAXSAnalysis software by an azimuthal integration of 330°.

The electrochemical active surface area (ECSA) of the gold nanostructured electrodes generated was assessed by cyclic voltammetry (CV) tests at a scan rate of 0.1 V s^−1^ and by linear sweep voltammetry (LSV), with the potential negatively swept from 1.5 V to 0.1 V versus Ag/AgCl in a 0.5 M sulfuric acid solution. 

The electrochemical active surface area (ECSA) determined by CV was divided by the geometric area of the Au disk electrode to obtain the roughness factor, RF (dimensionless).

### 2.3. Activity towards Glucose and Fuel Cell Set-Up and Operation

The activity of the templated Au nanostructured electrodes towards glucose oxidation in physiological pH conditions was assessed by chronoamperometry in a three-electrode set-up with Pt mesh as the counter electrode and Ag/AgCl (3 M KCl) as the reference electrode. The tests were performed at an applied potential of +0.22 V versus Ag/AgCl (3 M KCl) at increasing concentrations of glucose, ranging from 5 to 35 mM. For each concentration, the output steady-state current was recorded after 300 s and plotted against the glucose concentration. 

The Au nanostructured electrodes were also tested as the anode of an abiotic glucose fuel cell, with Pt wire (0.5 mm diameter) as the cathode. The electrodes were immersed into a beaker containing 6 mM glucose in 0.1 M phosphate buffer (pH 7.4) and connected to a PicoData Logger ADC-24 (Pico Technology, Eaton Socon, UK) to monitor the cell voltage. Initially, the GFC was operated in open circuit mode to allow the cell voltage to stabilize and reach a steady state. Subsequently, polarization experiments were conducted by applying varying external resistances to the system, ranging from 10,000,000 to 100 Ω, utilizing a Cropico resistor box (RS Components, Corby, UK). The power (P) profiles were derived from the polarization profiles, and calculated as P = V I, where V is the cell voltage (V) and I is the current (A). The internal resistance (R_int_) of the GFC was derived from the linear fit of the Ohmic region of the polarization curve. Both the power and current densities were normalised to the geometric area of the anode (0.0314 cm^2^). 

## 3. Result and Discussion

In this study, we explore the use of two templates to develop Au nanostructures. Figure 1 provides a schematic of the proposed role that these templates play in directing the nanostructure of the deposited gold. In the case of the Phytantriol/Brij^®^-56 template, we assume that several steps occur. First, the formation of a lipid membrane occurs (step i in Figure 1); the Phytantriol/Brij^®^-56 template forms a *Q*^*II*^_*D*_ triply periodic minimal surface at the electrode surface when immersed in an excess electrolyte solution. This structure is characterized by a continuous lipid bilayer that creates a highly ordered matrix. This step is followed by the channel formation (step ii in Figure 1); two sets of four-way non-intersecting aqueous channels are formed within the lipid matrix, separated by the lipid membrane. These channels confine the aqueous electrolyte, which effectively compartmentalizes the space through which electrodeposition can occur. Selective electrodeposition follows (step ii in Figure 1); the electrodeposition process is facilitated through one set of channels positioned closer to the bulk electrolyte. This selective deposition influences the gold nanostructure formation.

In the case of the hexane/SDS template, we assume that the surfactant (SDS) and the solvent (hexane) create micelles in the KCL solution which are randomly dispersed within the electrolyte solution. These micelles act as nano-containers that regulate the diffusion paths of Au ions. The presence of these micelles controls how Au ions travel towards and reach the electrode surface during the electrodeposition process. Due to the random yet regulated diffusion routes, Au ions are deposited at specific sites on the electrode surface, which are determined by the location and movement of the micelles.

**Figure 2:**
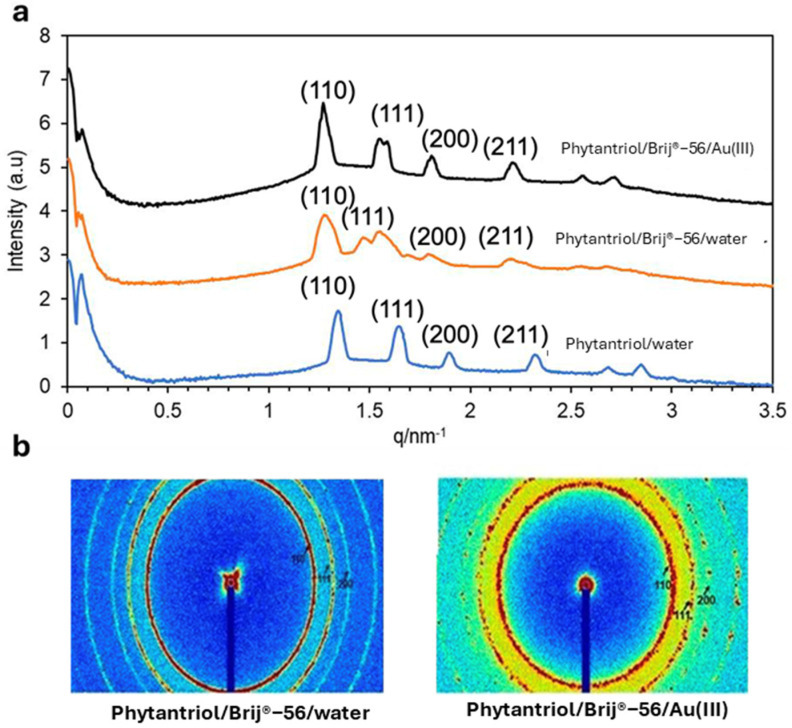
The 1D (**a**) and 2D (**b**) SAXS patterns obtained with the Phytantriol/Brij^®^−56 (80:20) solution, either in excess of water or in 0.01 M Au(III) 3 M KCl, or with Phytantriol in excess of water. The tests were performed at 25 °C.

Figure 2 shows the SAXS patterns (in both 1D and 2D) obtained with the Phytantriol and Brij^®^-56 template tested in this work at a temperature of 25 °C. Note that SAXS patterns were not obtained from the hexane/SDS template sample because, unlike the lipid cubic phase samples, they were not expected to show Bragg peaks.

The 1D and 2D SAXS patterns confirm that the Phytantriol and Brij^®^-56 created a lipid cubic phase in water. In the 1D graphs, the relative positions of the peaks follow the proportions √2:√3:√4 (expressed as ratios of 1/d and indexed as (hkl) = (110), (111), and (200)), which are in line with the first three reflections expected from a double-diamond cubic phase. An increase in the phytantriol cubic phase unit cell dimension of the lipid cubic phase template, from 6.5 to 7.0 nm, is observed in the presence of Brij^®^-56. This is attributed to a reduced lipid/aqueous interfacial curvature owing to the insertion of Brij^®^-56, and thus a more expanded unit cell is formed (see Figure 1a for a suggestion on the role of Brij^®^-56 in the Au electrodeposition process) [28]. The 2D SAXS patterns are composed of three sharp Debye–Scherrer rings that suggest the presence of well-defined cubic phase structures. The values of the lattice parameter calculated from SAXS patterns indicate that the presence of KCl and of Au (III) ions generated by the dissociation of hydrogen chloroauric acid do not impact the lipid and Brij^®^-56 headgroups and/or on the structure of the liquid crystal. Overall, the SAXS results suggest that the Phytantriol/Brij^®^-56 system can be a suitable soft-template candidate for the deposition of gold for the generation of controlled nanostructures. 

### 3.1. Electrode Characterisation

**Figure 3:**
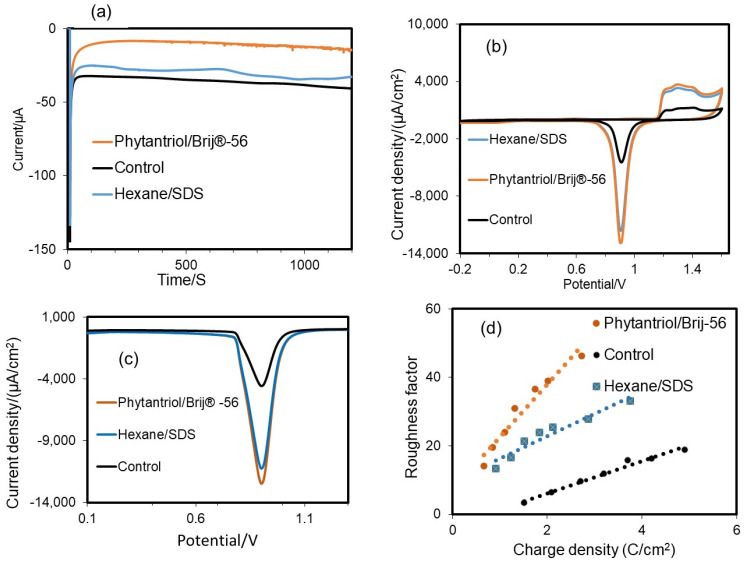
(**a**) Typical electrodeposition current recorded by the multi-step chronoamperometry technique for Au catalyst deposition on the disk electrode at a fixed potential of −0.25 V vs. Ag/AgCl, and assessment of the electrochemical active surface area (ECSA) and roughness factor of the gold nanostructured electrodes generated with the two soft-template methods tested in this study and with no template (control). (**b**) CV analysis in 0.5 M H_2_SO_4_ at a scan rate 0.1 V s^−1^. (**c**) Linear sweep voltammograms (LSV); the potential was negatively swept from 1.5 V to 0.1 V at a scan rate of 0.1 V s^−1^. (**d**) Variation in roughness factor with charge density for the Au nanostructured electrodes obtained with the two soft-templates and the control (no template).

A multi-step amperometry technique was used for the electrodeposition of Au in the presence and absence (the control) of a soft-based template. Figure 3a shows the amperometric response during Au electrodeposition. The highest transient current was observed at the beginning of the process for the control assay, suggesting faster electrodeposition on the surface of the bare electrode. The electrodeposition current at the beginning of the process is probably greater because of the higher diffusion of Au^3+^ ions. Steady-state currents were observed within 50 to 1000 s, due to the slow diffusion of Au (III) ions. The electrodeposition current was notably lower in the case of the Phytantriol/Brij^®^-56 template compared to the hexane/SDS template, as the size of the hydrophilic channels for Au electrodeposition in the former case was expected to be narrower.

CV tests were performed to assess the ECSA of the electrodes generated. As shown in Figure 3a, in all cases there was a noticeable and distinctive rapid rise in the current density on the anodic side, at around 1.20 V (Vs Ag/AgCl (3 N KCl) in 0.5 M sulfuric acid solution, due to the oxidation of Au [36]. When the potential scanning direction was reversed, a pronounced reduction in current density was observed at 1.6 V versus Ag/AgCl (3 N KCl), following a sharp reduction peak at 0.93 V versus Ag/AgCl (3 N KCl), in line with previous findings [37,38]. The reduction peak at 0.93 V vs. Ag/AgCl is reported to be related to the Au surface area at a ratio of 386 μC cm^−2^ [39]. This peak was very pronounced for the two electrodes generated with the soft-template method and it was much lower in the case of the electrode generated with no template. The resulting values of ECSA were 0.446 cm^2^ for the gold nanostructure obtained with the Phytantriol/Brij^®^-56 template and 0.421 cm^2^ for the gold nanostructure obtained with the Hexane/SDS template. These values were, respectively, 4.23 and 4.07 times higher than the value observed for the control (no use of template for the gold electrodeposition), which was 0.1035 cm^2^. A wide oxidation peak current was observed at around 1.16 V, which is aligned to previous findings for Au {111} surfaces [40]. The oxidation peak at +1.42 V indicates the presence of Au{100} facets with well-exposed step/kink locations [40]. This peak resulted to be more pronounced for the case of the Au nanostructures generated with the two soft-templates. This result suggests that these Au nanostructures may be characterised by an abundance of step/kink structures. 

To confirm the ECSA of the electrodes, LSV tests were performed (Figure 3b). The results showed that the ECSA values determined by LSV were equivalent to the values obtained by CV: 0.449 cm^2^ Au for the case of the Phytantriol/Brij^®^-56 soft-template; 0.4253 cm^2^ for the case of the hexane/SDS template; and 0.0936 cm^2^ for the case of no template. 

Figure 3c shows the variation in roughness factor with charge density for the different Au nanostructures. When more charge is applied and a thicker layer of gold is deposited onto the surface of the disk electrode, the roughness factor and consequently the active surface area increase proportionally.

**Figure 4:**
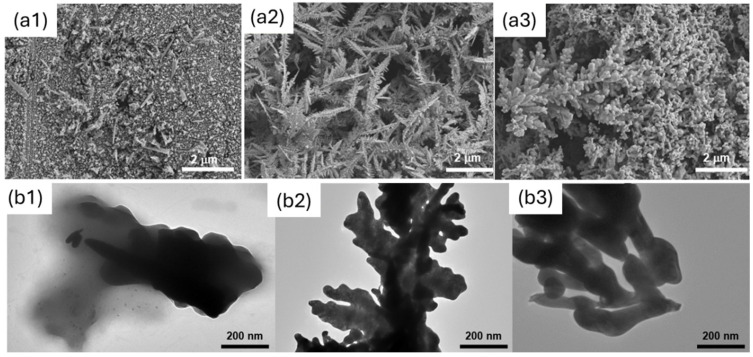
SEM (**a**) and TEM (**b**) analysis of the gold nanostructures generated. Here, (**a1**) and (**b1**) refer to the control (no template); (**a2**) and (**b2**) refer to Phytantriol/Brij^®^−56; and (**a3**) and (**b3**) refer to Hexane/SDS.

The morphology of the electrodes obtained with the two soft-template methods was investigated using SEM and TEM. The results were compared with the case of template-free electrodeposition (the control). Figure 4 shows the results obtained. As shown, the Phytantriol/Brij^®^-56 led to a hyperbranched structure that resembled nano-feathers with significant surface roughness along the edges, which were sharp and irregular, leading to a 3D structure with branch sizes ranging from 50 to 400 nm. The hexane/SDS template instead led to a nanocoral-like structure, characterised by smooth edges, with a 3D structure with particle sizes ranging from 50 to 300 nm. 

When no structure-directing agent is used, no 3D structure is observed, and the coverage of the electrode is poor and uneven.

We note that, although the use of structure-directing agents results in a morphology that is different from the control, the metal does not exactly adopt the nanostructure of the template in the case of the Phytantriol/Brij^®^-56 sample, as it did in previously reported studies using platinum, where the wires and pores had the same dimensions (<10 nm) as the aqueous channels of the Phytantriol/Brij^®^-56 lipid cubic phase template [28]. 

It was observed in previous studies that the ratio between Phytantriol and Brij^®^-56 in the soft-template can be modulated to tune the mesoporous metal nanowire networks resulting from the electrodeposition [28]. Accordingly, in future research it will be important to assess the impact that the composition of the template used has on the resulting gold nanostructures and, consequently on performance. 

### 3.2. Testing the Activity of the Nanostructured Au Electrodes toward Glucose

**Figure 5:**
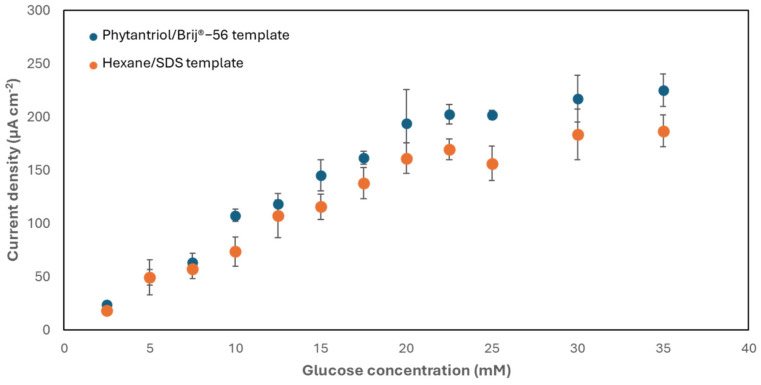
Chronoamperometry (+0.22 V versus Ag/AgCl, 3 M KCl) response of the two types of nanostructured gold electrode generated to increasing concentrations of glucose in phosphate buffer (0.1 M, pH 7.4). Error bars refer to a minimum of three replicates, each obtained with a different electrode.

The activity of the nanostructured Au electrodes developed with the two soft-template methods towards the electro-oxidation of glucose was subsequently assessed. For this purpose, the electrodes were exposed to increasing concentrations of glucose. Figure 5 shows the results obtained. As shown, the two electrodes exhibited the same trend, with a linear response to glucose within the range 2.5–20 mM and a sensitivity equal to 9.6 ± 1.5 μA mM^−1^ cm^−2^, with an upper limit of detection of 33 μM, for the electrode generated with the Phytantriol/Brij^®^-56 template, and equal to 7.8 ± 0.8 μA mM^−1^ cm^−2^, with an upper limit of detection of 48.2 μM, for the electrode generated with the hexane/SDS template. These values of sensitivity are greater than other studies, as documented in Table 1. These experiments were, however, conducted in an electrolyte (phosphate buffer; 0.1 M, pH 7.4) saturated with air, which resulted in a dissolved oxygen concentration exceeding the physiological range [41]. Research suggests that lower oxygen levels may reduce current outputs, highlighting the need for further investigation. Yet, the system can be engineered to enable the exposure of physiological fluids to air, potentially through the integration of air-permeable coatings [42].

### 3.3. Testing the Au Nanostructured Electrodes as the Anode of an Abiotic Glucose Fuel Cell

**Figure 6:**
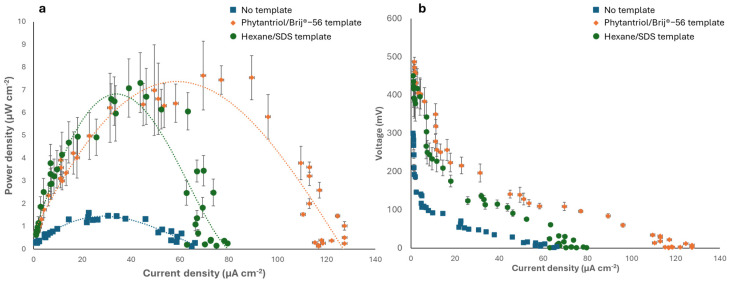
Electrochemical characterisation of the abiotic GFCs implementing the Au nanostructured anodes. (**a**) Power curves. (**b**) Polarisation curves. The tests were performed in 0.1 M phosphate buffer (0.1 M, pH 7.4) containing 6 mM glucose. Error bars refer to three replicates.

The Au nanostructured electrodes generated in this study were subsequently tested as the anode of an abiotic glucose fuel cell in a phosphate-buffered solution containing 6 mM glucose, with Pt wire as the cathode. Figure 6 shows the polarisation and power curves obtained and compares the results obtained with the case of a control anode electrode, generated with no template. As shown, the fuel cell with the Phytantriol/Brij^®^-56 templated anode exhibited the highest peak power density 7.6 μW cm^−2^ at a current density of 70 μA cm^−2^. The fuel cell with the Au nanocoral-like anode (hexane/SDS templated) generated a similar peak power, 7.3 μW cm^−2^, but the current density was much smaller, 43.7 μA cm^−2^. When the control anode (no template) was used, the peak power was approximately four times smaller (1.83 μW cm^−2^).

The GFC with the anode obtained from the two templated methods generated an open circuit voltage (OCV) of 487 ± 3.5 mV, in the case of the Phytantriol/Brij^®^-56 template, and 450 ± 5.5 mV in the case of the anode generated with the Hexane/SDS template. These values were nearly five times higher than the OCV generated by the GFC with the control anode, which was 100 ± 0.7 mV. The power output attained in this study is hindered by notable Ohmic and mass transport limitations, resulting in substantial internal resistance. The internal resistance of the GFCs was assessed by examining the Ohmic region of polarization curves. When the Phytantriol/Brij^®^-56 templated anode was used, an internal resistance of 35.7 KΩ was observed, whereas the hexane/SDS templated anode led to an internal resistance of 56.1 KΩ. Conversely, the GFC with the control anode exhibited an internal resistance of 98.1 KΩ. Overall, the power output observed in this research was comparable to the findings from previous studies on GFCs operating under similar oxygen and glucose concentrations [1,46]. Future research will involve testing the electrode performance in biological fluids, such as serum and interstitial fluids.

## 4. Conclusions

Abiotic glucose fuel cells can play a pivotal role in the development of miniaturised bioelectronics for the management of chronic conditions. In these systems, nanostructured gold is the preferred material choice for the anode electrode, due it its ability to catalyse the electrooxidation of glucose under neutral pH conditions. In this study, we explored the use of two soft-template methods in combination with electrodeposition for the creation of Au nanostructured electrodes: (i) a lipid cubic phase-based soft-template comprised of Phytantriol and Brij^®^-56 and (ii) an emulsion-based soft-template comprised of hexane and sodium dodecyl sulfate (SDS). Method (i) led to the generation of a nanofeather-like Au film, characterised by sharp edges, while method (ii) led to a nanocoral-like Au film, characterised by smooth and rounded edges. The resulting electrodes showed a similar electroactive surface area, which was found to be over four times higher than the value observed for the control, where no template was used for gold electrodeposition. When the activity towards glucose was assessed, the two nanostructured Au electrodes exhibited the same trend, with a linear response to glucose within the range 2.5–20 mM and a sensitivity equal to 9.6 ± 1.5 μA mM^−1^ cm^−2^ for the Phytantriol/Brij^®^-56 templated electrode and to 7.8 ± 0.8 μA mM^−1^ cm^−2^ for the hexane/SDS templated electrode. Finally, when tested as the anode of a GFC, the Au nanostructured electrodes led to a similar peak power density of over 7 μW cm^−2^ in phosphate augmented with 6 mM glucose, but in the case of the anode generated with the Phytantriol/Brij^®^-56 template the current density was almost twice higher, reaching the value of 70 μA cm^−2^. Overall, this study demonstrated a simple, cost-effective and successful route for the generation of controlled Au nanostructures for the electro-oxidation of glucose with promising fuel cell applications for wearable and implantable energy harvesting.

## Figures and Tables

**Table 1:** Comparison of glucose sensitivity of our current study with the literature reports.

Anode Material	Cathode Material	Linear Range (mM)	Condition	Sensitivity (μA mM^−1^ cm^−2^)	Ref.
Highly porous Au	Highly porous Au	0.01–10	34 mM PBS solution, pH 7.4	3.1	[14]
Highly porous Au	Pt catalyst	0.3–9	100 mM PBS solution, pH 7.4	8.8	[3]
Mesoporous Pt	Pt catalyst	0−10	100 mM PBS solution, pH 7.4	9.6	[43]
Ordered Pt nanotubule arrays	Pt	2–14	50 mM PBS solution, pH 7.4, 0.1 M KCl	0.1	[44]
Pt nanoflower-SWCNT membrane	Pt disk electrode	0.002–10	100 mM PBS solution, pH 7.4	7.26	[45]
Au nanotube arrays	Pt platinum wire	1–42.5	100 mM PBS solution, pH 7.4	1.13	[11]
Nanocoral-like Au film	Pt flag	2.5–20	100 mM PBS solution, pH 7.4	7.8 ± 0.8	this study
Nanofeather like Au film	Pt flag	2.5–20	100 mM PBS solution, pH 7.4	9.6 ± 1.5	this study

## Data Availability

Data are available upon request.

## References

[1] Gonzalez-Solino C., Bernalte E., Bayona Royo C., Bennett R., Leech D., Di Lorenzo M. (2021). Self-Powered Detection of Glucose by Enzymatic Glucose/Oxygen Fuel Cells on Printed Circuit Boards. ACS Appl. Mater. Interfaces.

[2] Antolini E. (2021). External abiotic glucose fuel cells. Sustain. Energy Fuels.

[3] Gonzalez-Solino C., Bernalte E., Metcalfe B., Moschou D., Di Lorenzo M. (2020). Power generation and autonomous glucose detection with an integrated array of abiotic fuel cells on a printed circuit board. J. Power Sources.

[4] Zhiani M., Barzi S., Ahmadi A., Vizza F., Gharibi H., Azhari A. (2022). Ex vivo energy harvesting by a by-pass depletion designed abiotic glucose fuel cell operated with real human blood serum. J. Power Sources.

[5] Jiang H., Akita T., Ishida T., Haruta M., Xu Q. (2011). Synergistic Catalysis of Au@Ag Core—Shell Nanoparticles Stabilized. J. Am. Chem. Soc..

[6] Hong W., Wang J., Wang E. (2014). Dendritic Au/Pt and Au/PtCu nanowires with enhanced electrocatalytic activity for methanol electrooxidation. Small.

[7] Henning A.M., Watt J., Miedziak P.J., Cheong S., Santonastaso M., Song M., Takeda Y., Kirkland A.I., Taylor S.H., Tilley R.D. (2013). Gold–palladium core–shell nanocrystals with size and shape control optimized for catalytic performance. Angew. Chem. Int. Ed..

[8] Zhang Y., Shen W., Kuang W., Guo S., Li Y., Wang Z. (2017). Serrated Au/Pd Core/Shell Nanowires with Jagged Edges for Boosting Liquid Fuel Electrooxidation. ChemSusChem.

[9] Lu F., Zhang Y., Liu S., Lu D., Su D., Liu M., Zhang Y., Liu P., Wang J.X., Adzic R.R. (2017). Surface Proton Transfer Promotes Four-Electron Oxygen Reduction on Gold Nanocrystal Surfaces in Alkaline Solution. J. Am. Chem. Soc..

[10] Tohidi M., Mahyari F.A., Safavi A. (2015). A seed-less method for synthesis of ultra-thin gold nanosheets by using a deep eutectic solvent and gum arabic and their electrocatalytic application. RSC Adv..

[11] Zhou Y.-G., Yang S., Qian Q.-Y., Xia X.-H. (2009). Gold nanoparticles integrated in a nanotube array for electrochemical detection of glucose. Electrochem. Commun..

[12] Othman A., Bilan H.K., Katz E., Smutok O. (2022). Highly Porous Gold Electrodes—Preparation and Characterization. ChemElectroChem.

[13] Banerjee S., Slaughter G. (2022). A tattoo-like glucose abiotic biofuel cell. J. Electroanal. Chem..

[14] du Toit H., Di Lorenzo M. (2014). Electrodeposited highly porous gold microelectrodes for the direct electrocatalytic oxidation of aqueous glucose. Sens. Actuators B Chem..

[15] Shin H.J., Ryoo R., Liu Z., Terasaki O. (2001). Template synthesis of asymmetrically mesostructured platinum networks. J. Am. Chem. Soc..

[16] Yamauchi Y., Kuroda K. (2008). Rational design of mesoporous metals and related nanomaterials by a soft-template approach. Chem.-Asian J..

[17] Zhang J., Li C.M. (2012). Nanoporous metals: Fabrication strategies and advanced electrochemical applications in catalysis, sensing and energy systems. Chem. Soc. Rev..

[18] Wang Q., Shantz D.F. (2008). Ordered mesoporous silica-based inorganic nanocomposites. J. Solid State Chem..

[19] Takai A., Doi Y., Yamauchi Y., Kuroda K. (2010). Soft-chemical approach of noble metal nanowires templated from mesoporous silica (SBA-15) through vapor infiltration of a reducing agent. J. Phys. Chem. C.

[20] Kuroda Y., Yamauchi Y., Kuroda K. (2010). Integrated structural control of cage-type mesoporous platinum possessing both tunable large mesopores and variable surface structures by block copolymer-assisted Pt deposition in a hard-template. Chem. Commun..

[21] Kang Y., Tang Y., Zhu L., Jiang B., Xu X., Guselnikova O., Li H., Asahi T., Yamauchi Y. (2022). Porous Nanoarchitectures of Nonprecious Metal Borides: From Controlled Synthesis to Heterogeneous Catalyst Applications. ACS Catal..

[22] Attard G.S., Corker J.M., Göltner C.G., Henke S., Templer R.H. (1997). Liquid-Crystal Templates for Nanostructured Metals. Angew. Chem. Int. Ed..

[23] Li C., Iqbal M., Lin J., Luo X., Jiang B., Malgras V., Wu K.C.-W., Kim J., Yamauchi Y. (2018). Electrochemical Deposition: An Advanced Approach for Templated Synthesis of Nanoporous Metal Architectures. Accounts Chem. Res..

[24] Wang Y., He C., Xing W., Li F., Tong L., Chen Z., Liao X., Steinhart M. (2010). Nanoporous metal membranes with bicontinuous morphology from recyclable block-copolymer templates. Adv. Mater..

[25] Takai A., Yamauchi Y., Kuroda K. (2010). Tailored electrochemical synthesis of 2d-hexagonal, lamellar, and cage-type mesostructured pt thin films with extralarge periodicity. J. Am. Chem. Soc..

[26] Attard G.S., Bartlett P.N., Coleman N.R.B., Elliott J.M., Owen J.R., Wang J.H. (1997). Mesoporous platinum films from lyotropic liquid crystalline phases. Science.

[27] Akbar S., Elliott J.M., Rittman M., Squires A.M. (2013). Facile production of ordered 3D platinum nanowire networks with “single diamond” bicontinuous cubic morphology. Adv. Mater..

[28] Akbar S., Boswell J., Waters S., Williams S., Elliott J.M., Squires A.M. (2021). Control of Pore and Wire Dimensions in Mesoporous Metal Nanowire Networks through Curvature Modulation in Lipid Templates: Implications for Use as Electrodes. ACS Appl. Nano Mater..

[29] Iqbal M., Li C., Wood K., Jiang B., Takei T., Dag Ö., Baba D., Nugraha A.S., Asahi T., Whitten A.E. (2017). Continuous Mesoporous Pd Films by Electrochemical Deposition in Nonionic Micellar Solution. Chem. Mater..

[30] Wang H., Wang L., Sato T., Sakamoto Y., Tominaka S., Miyasaka K., Miyamoto N., Nemoto Y., Terasaki O., Yamauchi Y. (2012). Synthesis of mesoporous Pt films with tunable pore sizes from aqueous surfactant solutions. Chem. Mater..

[31] Li C., Dag Ö., Dao T.D., Nagao T., Sakamoto Y., Kimura T., Terasaki O., Yamauchi Y. (2015). Electrochemical synthesis of mesoporous gold films toward mesospace-stimulated optical properties. Nat. Commun..

[32] Baba D., Kim J., Henzie J., Li C., Jiang B., Dag Ö., Yamauchi Y., Asahi T. (2018). Electrochemical deposition of large-sized mesoporous nickel films using polymeric micelles. Chem. Commun..

[33] Li C., Jiang B., Chen H., Imura M., Sang L., Malgras V., Bando Y., Ahamad T., Alshehri S.M., Tominaka S. (2016). Superior electrocatalytic activity of mesoporous Au film templated from diblock copolymer micelles. Nano Res..

[34] Akbar S., Elliott J.M., Squires A.M., Anwar A. (2020). Optimum conditions for electrochemical deposition of 3-D mesoporous platinum framework. J. Nanopart. Res..

[35] Akbar S., Boswell J., Worsley C., Elliott J.M., Squires A.M. (2018). Ultrathin Uniform Platinum Nanowires via a Facile Route Using an Inverse Hexagonal Surfactant Phase Template. Langmuir.

[36] Shiba S., Hirabayashi S., Niwa O., Kato D., Kunitake M., Matsuguchi M. (2020). Monolithic Au Nanoscale Films with Tunable Nanoporosity Prepared via Dynamic Soft Templating for Electrocatalytic Oxidation of Methanol. ACS Appl. Nano Mater..

[37] Kumari R., Chandra P. (2023). Electrochemical Nano-Imprinting of Trimetallic Dendritic Surface for Ultrasensitive Detection of Cephalexin in Pharmaceutical Formulations. Pharmaceutics.

[38] Henning S., Herranz J., Gasteiger H.A. (2015). Bulk-Palladium and Palladium-on-Gold Electrocatalysts for the Oxidation of Hydrogen in Alkaline Electrolyte. J. Electrochem. Soc..

[39] Burke L.D., Nugent P.F. (1997). The electrochemistry of gold: I the redox behaviour of the metal in aqueous media. Gold Bull..

[40] Chauhan P., Hiekel K., Diercks J.S., Herranz J., Saveleva V.A., Khavlyuk P., Eychmüller A., Schmidt T.J. (2021). Electrochemical Surface Area Quantification, CO_2_ Reduction Performance, and Stability Studies of Unsupported Three-Dimensional Au Aerogels versus Carbon-Supported Au Nanoparticles. ACS Mater. Au.

[41] Ortiz-Prado E., Dunn J.F., Vasconez J., Castillo D., Viscor G. (2019). Partial pressure of oxygen in the human body: A general review. Am. J. Blood Res..

[42] Oncescu V., Erickson D. (2013). High volumetric power density, non-enzymatic, glucose fuel cells. Sci. Rep..

[43] Park S., Chung T.D., Kim H.C. (2003). Nonenzymatic glucose detection using mesoporous platinum. Anal. Chem..

[44] Yuan J.H., Wang K., Xia X.H. (2005). Highly ordered platinum-nanotubule arrays for amperometric glucose sensing. Adv. Funct. Mater..

[45] Su L., Jia W., Zhang L., Beacham C., Zhang H., Lei Y. (2010). Facile synthesis of a platinum nanoflower monolayer on a single-walled carbon nanotube membrane and its application in glucose detection. J. Phys. Chem. C.

[46] Shen F., Pankratov D., Halder A., Xiao X., Toscano M.D., Zhang J., Ulstrup J., Gorton L., Chi Q. (2019). Two-dimensional graphene paper supported flexible enzymatic fuel cells. Nanoscale Adv..

